# Prevalence and concentration of *stx*+ *E*. *coli* and *E*. *coli* O157 in bovine manure from Florida farms

**DOI:** 10.1371/journal.pone.0217445

**Published:** 2019-05-24

**Authors:** Christopher A. Baker, Jaysankar De, Bruna Bertoldi, Laurel Dunn, Travis Chapin, Michele Jay-Russell, Michelle D. Danyluk, Keith R. Schneider

**Affiliations:** 1 Department of Food Science and Human Nutrition, University of Florida, Gainesville, Florida, United States of America; 2 Citrus Research and Education Center, University of Florida, Lake Alfred, Florida, United States of America; 3 Western Center for Food Safety, University of California, Davis, California, United States of America; University of Delhi, INDIA

## Abstract

Fresh produce outbreaks due to Shiga toxin-producing *Escherichia coli* (STEC) continue to occur in the United States (US). Manure-amended soils can pose a public health risk when used for growing raw agricultural commodities. Knowing the prevalence and concentration of STEC in untreated biological soil amendments of animal origin (BSAAO) is important to help guide the most appropriate pre-harvest interval(s) following application to limit risks from these soil amendments. Bovine manure samples were collected from 12 farms in Florida, including samples from piles, lagoons, barns, and screened solids. Two methods were used to detect *stx1*/*2* and *rfbE* genes in samples. A prevalence rate of 9% for *stx1* and/or *stx2* and 19% for *rfbE* was observed from the 518 bovine manure samples evaluated. A most probable number (MPN) assay was performed on *stx*+ samples when applicable. The geometric mean for *stx+* samples (n = 20) was 3.37 MPN g^-1^ (0.53 log MPN g^-1^) with a maximum value of 6,800 MPN g^-1^ (3.83 log MPN g^-1^). This research was part of a larger nationwide geographical study on the prevalence and concentration of STEC in bovine manure to help guide regulations on feasible pre-harvest intervals for the application of untreated BSAAO.

## Introduction

Outbreaks due to Shiga toxin-producing *Escherichia coli* (STEC) are costly to the economy and can affect both the public sector as well as growers/shippers in the produce industry. It has been estimated that over 265,000 illnesses occur annually in the United States (US) due to STEC, with over 3,600 hospitalizations and 30 deaths [1]. In 2018, it was reported that confirmed and suspected foodborne incidences of STEC accounted for 203 (5%) outbreaks, 2,465 (3%) illnesses, 693 (13%) hospitalizations, and 13 (9%) deaths from 2009 to 2015 in the US (2). These figures include all food types, though produce-related outbreaks represent a substantial portion of the STEC foodborne outbreaks in the US [2, 3]. From 2010 to 2015, STEC caused 48 known produce-related outbreaks and 942 known cases in the US, with a median of 15 cases per produce-related outbreak during this period [4]. A STEC outbreak linked to spinach in 2006 resulted in 199 illnesses, 102 hospitalizations, and three deaths [5]. In 2018, a STEC outbreak linked to romaine lettuce exceeded the case count of 2006, resulting in 210 illnesses, 96 hospitalizations, and five deaths [6].

There are many potential routes of produce contamination that may lead to foodborne illness, one of which is contamination through the application of untreated manure to soils. Application of bovine manure to soil can benefit both livestock/dairy and produce farmers. Manure is a commonly added organic soil amendment used to prevent soil erosion, replenish nutrients within the soil, and maintain soil quality each growing season following the repeated use of agricultural lands [7]. However, soils amended with untreated BSAAO present a microbial food safety risk [8, 9], especially if these amendments are not handled, transported, stored, and applied properly [10].

In the US Food Safety Modernization Act (FSMA), the Produce Safety Rule (PSR) has been set forth to regulate the growing of fresh produce [11]. Subpart F of the PSR focuses on the use of biological soil amendments of animal origin (BSAAO), which is defined as “a biological soil amendment which consists, in whole or in part, of materials of animal origin, such as manure or non-fecal animal byproducts including animal mortalities, or table waste, alone or in combination” [12]. In 2013, it was proposed that farmers would be required to wait nine months after application of untreated BSAAO to harvest produce [10]. This resulted in negative feedback from farmers [10], and consequently the minimum harvest interval for untreated BSAAO is reserved until a feasible and data-driven harvest interval can be set [12]. Previous research on STEC prevalence in bovine manure is available, but few studies assessing STEC concentrations in manure are available [13]. In this study, *stx* (*stx1* and *stx*2) and *rfbE* (specific for *E*. *coli* O157) were targeted to obtain prevalence data in manure samples. Most probable number (MPN) g^-1^ values were determined for *stx*+ samples. Samples which were presumptive positive for *stx* were further screened for *eae* to determine the prevalence for atypical enteropathogenic *E*. *coli* (aEPEC).

The *stx* genes are major virulence factors of STEC and thus are the primary genes targeted for molecular STEC detection assays [14]. The *eae* gene, which codes for the intestinal adherence factor intimin, is also commonly used to screen for STEC [15]. The *rfbE* gene specific for O157 lipopolysaccharide has also been established as a molecular marker to detect *E*. *coli* O157 [15, 16]. *E*. *coli* O157 is the major STEC serogroup known to cause illness and has been associated with numerous produce-related foodborne outbreaks and is commonly associated with severe complications in illness [17–20].

The prevalence of STEC in bovine manure has been assessed in previous studies, with a wide range of prevalence rates documented in beef and dairy production systems [17, 21–22]. Despite previous research, an assessment of multiple farms in several geographical regions for *stx* and *rfbE* presence in bovine manure was warranted. The objectives of this research project were to determine the prevalence and concentration of STEC, and the prevalence of *E*. *coli* O157 in bovine manure on Florida livestock and dairy farms.

## Materials and methods

### Sample collection and non-selective enrichment

Manure samples were collected from a total of 74 bovine manure storage locations at 12 farms (10 dairy farms, one beef feedlot, and one livestock market). Each farm was sampled twice from August 2017 to May 2018. Farm visits were made based on the willingness and availability of the farmers participating in the study. Three to four storage locations were sampled per farm; storage locations include piles (aggregated, stacked manure), lagoon (liquid manure storage basin), barn (unmoved, unstacked manure), and/or screened solid (dewatered manure) samples. Seven samples were collected from each storage location. Surface or subsurface samples were obtained for all pile and screened solid samples, and for barn samples when applicable. The following information was collected from each farm: farm type (beef, dairy, livestock market), sample temperature, sample depth, storage location, storage age, time of manure pile sitting undisturbed, pile size, degree of protection from birds, equipment cleaning frequency, and any potential external influences on the pile when applicable. Relative humidity, wind speed, and precipitation (past 24 h) were obtained from the Florida Automated Weather Network (FAWN) (http://fawn.ifas.ufl.edu).

A total of 518 bovine manure samples were evaluated. Thirty grams of sample were added to 270 mL of tryptic soy broth (TSB; Becton, Dickinson and Company, Sparks, MD) in 24 oz. Whirl-Pak sterile filter (0.33 mm) bags (Teel Plastics, Inc., Baraboo, WI) to keep liquid separate from solids. Each sample bag was rubbed by hand for 1 min to obtain a mixed manure-TSB slurry. Bags with the manure-TSB slurry were incubated in a GYROMAX 747 orbital shaking incubator (Amerex Instruments, Inc., Lafayette, CA) for 2 h at 25°C, 100 rpm followed by 8 h at 42°C, 100 rpm.

### Selective enrichment for STEC (mEHEC method)

**Fig 1:**
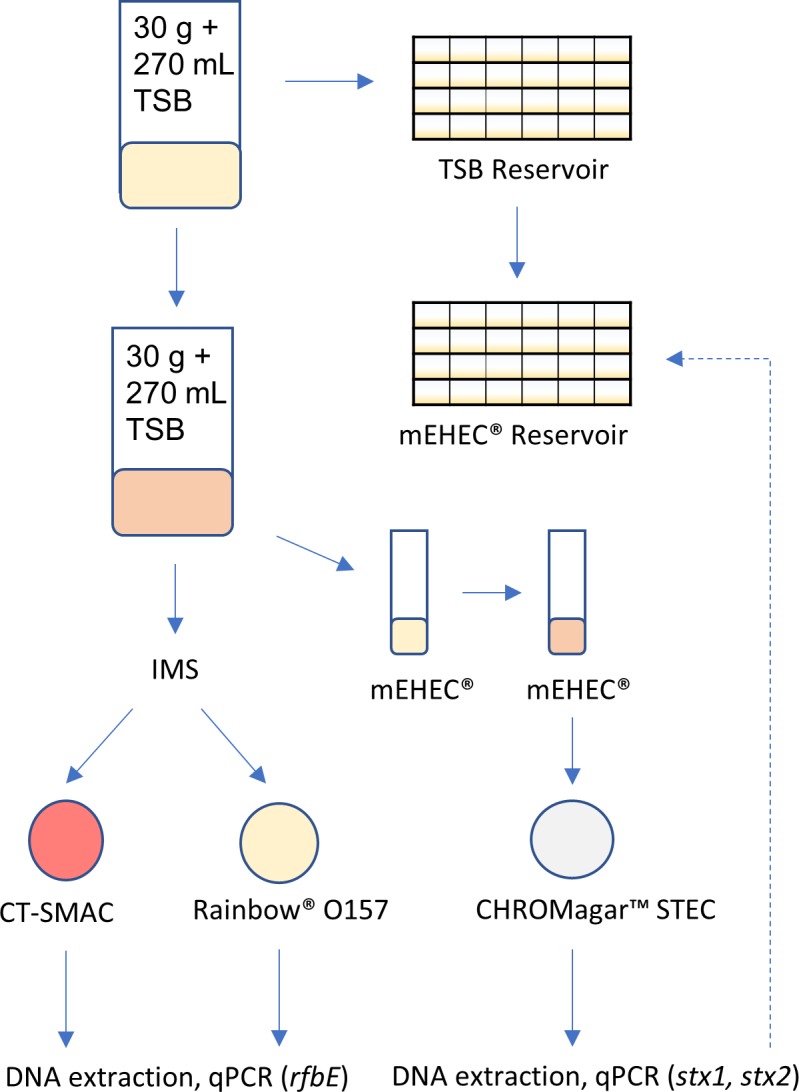
Flow diagram of mEHEC and IMS-O157 methods. The corresponding mEHEC reservoirs for *stx*+ samples were screened for *stx* to obtain an MPN g^-1^ value.

Once sample bags (30 g + 270 mL of TSB) were incubated, 1 mL of manure-TSB slurry was added to 9 mL of mEHEC (Biocontrol, Bellevue, WA) broth and incubated for 12 h at 42°C [23, 24]. Enrichments in mEHEC broth were streaked for isolation on CHROMagar STEC (CHROMagar Microbiology, Paris, France) and incubated for 24 h at 37°C (Fig 1). Up to 12 presumptive positive colonies on CHROMagar STEC were re-streaked until pure colonies were obtained.

### DNA extraction

A loopful of presumptive positive colonies were added to a sterile 1.5 mL centrifuge tube containing 100 μL of DNA grade water (Thermo Fisher Scientific, Fair Lawn, NJ), and vortexed for 10 s. Tubes were heated at 100°C for 20 min, and centrifuged for 10 min at 12,000 x g. Following centrifugation, supernatant with DNA was obtained and stored at -20°C until further analysis.

### Detection of *stx1* and *stx2* by quantitative PCR

DNA from presumptive positive colonies was screened for *stx1* and *stx2*. Quantitative PCR (qPCR) was performed on a Bio-Rad iCycler Optical Model (Bio-Rad, Hercules, CA) in a 20 μL reaction with 2 μL of DNA, 10 μL iTaq universal probes supermix (Bio-Rad), 0.3 μM of each primer and 0.25 μM of each probe, and 7.6 μL of sterile DNA grade water. The qPCR reaction was performed under the following conditions: 95°C for 20 s, 40 cycles of 95°C for 3 s, 60°C for 30 s, followed by a 4°C hold. The *stx1* and *stx2* primer and probe sequences (Table 1) used in this analysis were obtained from Cooley et al. [25] and synthesized by IDT (Integrated DNA Technologies, Inc., Skokie, IL). If a sample was positive for *stx1* and/or *stx2*, the respective mEHEC reservoirs were evaluated for *stx1* and *stx2* by qPCR to determine the MPN g^-1^ for the *stx1* and/or *stx2* positive manure sample.

**Table 1:** Primers and probes for the detection of *stx1*, *stx2*, *rfbE* and *eae* genes.

Primer	Sequence	Reference
*stx1*-f	CAT-CGC-GAG-TTG-CCA-GAA-T	[26]
*stx1*-r	TCC-CAC-GGA-CTC-TTC-CAT-CT	[26]
*stx1*-p	/56-FAM/ATC TGA TGA/ZEN/TTT CCT TCT ATG TGT CCG/3IABkFQ/	[26]
*stx2-*f	GGA-CCA-CAT-CGG-TGT-CTG-TTA-TT	[26]
*stx2-*r	CCC-TCG-TAT-ATC-CAC-AGC-AAA-AT	[26]
*stx2*-p	/56-JOEN/CCA CAC CCC/ZEN/ACC GGC AGT 3IABkFQ/	[26]
*rfbE*-f	CTG-TCC-ACA-CGA-TGC-CAA-TG	[59]
*rfbE*-r	CGA-TAG-GCT-GGG-GAA-ACT-AGG	[59]
*rfbE*-p	/56-FAM/TTA-ATT-CCA-CGC-CAA-CCA-AGA-TCC-TCA/3IABkFQ/	[59]
*eae*-f	AAA-GCG-GGA-GTC-AAT-GTA-ACG	[15]
*eae*-r	GGC-GAT-TAC-GCG-AAA-GAT-AC	([15]
*eae*-p*	/5HEX/CTC-TGC-AGA-TTA-ACC-TCT-GCC-G/3BHQ_1/	[15]

*Probe fluorophore and quencher modified from Noll et al. [15].

### Sample preparation for potential positive samples and MPN g^-1^ analysis

Prior to the initial incubation of the sample bags (30 g + 270 mL of TSB), 5.5 mL of the slurry was transferred to each of the first four rows (four replicates) of the first column of a reservoir (4 X 6 wells). The first column thus only received the slurry, while columns 2–6 were filled with 4.5 mL of TSB for serial dilution. Once samples were added to the first column of the respective reservoirs, 10-fold serial dilutions were performed in columns 2–6 by transferring 0.5 mL from the previous column to the adjacent columns with a multi-channel pipette. After reservoirs were incubated simultaneously with the sample bags, 0.5 mL of incubated TSB broth from each well was transferred to respective wells in separate reservoirs containing 4.5 mL of mEHEC broth in each well. The mEHEC reservoirs were incubated simultaneously with the mEHEC tubes for 12 h at 42°C and held at 6°C until *stx* presence was determined following mEHEC tube analysis. This scheme provided a 4 X 6 dilution MPN reservoir to enumerate any positive samples.

### Determination of the MPN g^-1^ of *stx1* and/or *stx2*+ samples

Once a *stx1* and/or *stx2*+ sample was confirmed by qPCR, the corresponding mEHEC reservoirs from *stx1*/*stx2*+ samples were further evaluated to determine the MPN g^-1^ value. An aliquot was drawn from each of the 24 wells with a multi-channel pipette and streaked onto CHROMagar STEC, and plates were incubated as previously described. Colonies were isolated, and DNA was extracted and evaluated for *stx1*/*stx2* via qPCR as previously described. Once *stx* presence was determined for each well, MPN calculations were performed using an MPN calculator [26] to determine the MPN g^-1^ value. For MPNs with all 24 wells negative, the single positive from the 30 g sample was used to calculate the MPN g^-1^, which resulted in a limit of detection (LOD) of 0.089 MPN g^-1^ (-1.05 log MPN g^-1^).

### Immunomagnetic separation (IMS) and isolation of *E*. *coli* O157 (IMS-O157 method)

Following the incubation of TSB bags as previously described, 1 mL of enrichment was added to a 1.5 mL tube containing 20 μL of DynabeadsMAX anti *E*. *coli* O157 (Invitrogen, Frederick, MD) and vortexed for 10 s. Tubes were placed in an MPC-S rack, inverted several times, and incubated at room temperature for 10 min with gentle agitation. Following incubation, a magnetic plate was inserted into the MPC-S rack, inverted several times, and incubated for 3 min. Sample supernatant was aspirated, and the magnetic plate was removed from the MPC-S rack. One mL of 1x wash buffer (Invitrogen, Frederick, MD) was added to each tube, inverted several times, and incubated for 3 min with the magnetic plate. The wash step was repeated twice, and after the third wash the beads were re-suspended in 100 μL of wash buffer. Re-suspended cells were streaked on BBLMacConkey II Agar with Sorbitol (Becton, Dickinson and Company, Sparks, MD) supplemented with cefixime (0.05 μg/mL) (US Pharmacopeia, Rockville, MD) and potassium tellurite (2.5 μg/mL) (Chem-Impex International, Inc., Wood Dale, IL) (CT-SMAC) and Rainbow Agar O157 (BIOLOG, Inc., Hayward, CA). Plates were incubated for 24 h at 37°C, and presumptive positive *E*. *coli* O157 colonies on CT-SMAC and Rainbow Agar O157 [27] were re-streaked on respective plates and incubated for 24 h at 37°C.

### Quantitative PCR on *E*. *coli* O157 and additional analyses

DNA extraction was performed as previously described from isolated presumptive positive colonies and stored at -20°C until further analysis. Quantitative PCR was performed following the methods described in Jacob et al. [28] on a Bio-Rad iCycler Optical Model (Bio-Rad). The 20 μL PCR reaction consisted of 2 μL of DNA, 10 μL iTaq universal probes supermix (Bio-Rad), 0.5 μM of primers and probes, and 5 μL of sterile DNA grade water and was performed under the following conditions: 95°C for 10 min, 45 cycles of 95°C for 15 s, 56°C for 20 s, 72°C for 40 s, followed by a 4°C hold. The primers and probe targeting the *rfbE* gene were obtained from Jacob et al. [28] and are available in Table 1. Sample DNA that was positive for *E*. *coli* O157 was screened for *stx1* and *stx2* via qPCR to determine if positive *E*. *coli* O157 isolates also contained *stx1* and/or *stx2*. Sample DNA from the mEHEC and IMS-O157 methods was screened for *eae* presence via qPCR, and primers and probes were obtained from Noll et al. [15] and are listed in Table 1.

### Statistical analysis

Prevalence was determined as the number of positive samples for each gene divided by the total number of samples evaluated. A two-sided Wilcoxon test was performed to compare the effect of sample temperature and sample depth on the presence of *stx* and *rfbE*. Nonparametric correlation tests (Spearman) were used to compare log transformed MPN g^-1^ values with sample temperature and depth. Fisher’s exact tests adjusted with Hommel’s modification of the Bonferroni procedure were performed for *stx*, *stx1*, *stx2*, and *rfbE* presence based on storage location, pile age, and for surface versus subsurface samples. A significance level (α) of 0.05 was used for all analyses. All statistical analyses were performed using R version 3.4.3 (http://www.R-project.org).

## Results

### Prevalence of *stx1*/*stx2* and *rfbE* in manure samples

Of the 518 samples evaluated, 20 (4%) *stx1* and/or *stx2* positive samples were identified in the mEHEC method alone. There were 97 (19%) *rfbE*+ samples identified with the IMS-O157 method. Sample DNA that was *rfbE*+ was further analyzed for the presence of *stx1* and *stx2*, and 26 additional *stx*+ samples were identified. Including the mEHEC and IMS-O157 methods, *stx1* and/or *stx2* was detected in 46 samples (9% prevalence) (12 *stx1+/stx2+*; eight *stx1+/stx2-*; and 26 *stx1-/stx2+)*. This resulted in a prevalence of 4% (20/518) for *stx1* and 7% (38/518) for *stx2*. The prevalence for *stx1/2* in samples increased by a factor of 2.3 when combining both methods, and all data analyses included *stx*+ samples from both methods.

Of the 74 storage locations tested, 26 (35%) were *stx1* and/or *stx2* positive, and 43 (58%) were *rfbE*+. Of the 12 farms and 24 farm visits, 11 farms (92%) and 15 farm visits (63%) resulted in a *stx*+ sample, and *stx* was found during both visits for four farms (33%). Only one of the 12 farms (8%) was *stx*- on both visits. Twelve farms (100%) and 23 of 24 farm visits (96%) resulted in at least one *rfbE*+ sample.

### Prevalence of *eae* in bovine manure samples

There were seven *eae*+ samples detected among the 20 *stx1/2*+ samples originally identified in the mEHEC method, and 26 additional *eae*+ samples detected among the 27 *stx*+ samples identified following the IMS-O157 method. This resulted in a total of 10 *stx1*+/*stx2*+/*eae*+ samples; four *stx1*+/*stx2*-/*eae*+ samples; 19 *stx1*-/*stx2*+/*eae*+ samples; two *stx1*+/*stx2*+/*eae*- samples; six *stx1*+/*stx2*-/*eae*- samples; and nine *stx1*-/*stx2*+/*eae*- samples. Samples negative for *stx* were evaluated for *eae*, which resulted in a total of 28 *stx1*-/*stx2*-/*eae*+ samples, 14 (50%) of which were from a single farm visit, and 13 (46%) were from a single farm found during the two visits. Of the 97 *rfbE*+ samples, there were 27 *stx*+/*eae*+, two *stx*+/*eae*-, 12 *stx*-/*eae*+, and 56 *stx*-/*eae*- samples.

### Most probable number *stx*+ values in bovine manure samples

Of the 20 *stx+* samples evaluated by the MPN assay, the geometric mean was 3.37 MPN g^-1^ (0.53 log MPN g^-1^) with a maximum value of 6,800 MPN g^-1^ (3.83 log MPN g^-1^) (Table 2). Fifteen of the 20 MPN values (75%) were below 23 MPN g^-1^ (<1.36 log MPN g^-1^). Of the 20 MPN values, four were *rfbE*+ (Table 2).

**Table 2:** MPN g-^1^ values for *stx*+ bovine manure samples with 95% confidence intervals.

Manure Type	Storage Location	*stx1*	*stx2*	*rfbE*	*eae*	MPN g^-1^*	LL**	UL**	
Feedlot	Lagoon	+	-	-	-	220 (2.34)	71	710	
Livestock Market	Pile	+	+	-	-	6800 (3.83)	N/A	N/A	
Livestock Market	Pile	+	+	+	+	110 (2.04)	31	390	
Livestock Market	Pile	+	-	-	-	1.2 (0.08)	0.3	4.8	
Livestock Market	Pile	-	+	-	-	0.91 (-0.04)	0.2	4.2	
Dairy	Lagoon	-	+	-	-	1.6 (0.20)	0.45	5.6	
Dairy	Lagoon	-	+	-	-	0.089 (-1.05)	0.009	0.89	
Dairy	Pile	+	-	+	+	0.089 (-1.05)	0.009	0.89	
Dairy	Pile	+	+	-	-	23 (1.36)	7.4	72	
Dairy	Screened solids	-	+	-	-	47 (1.67)	16	140	
Dairy	Screened solids	-	+	-	-	9.6 (0.98)	2.8	33	
Dairy	Screened solids	-	+	-	-	5.5 (0.74)	1.8	17	
Dairy	Screened solids	-	+	-	-	3.3 (0.52)	1.1	9.7	
Dairy	Screened solids	-	+	+	+	0.45 (-0.35)	0.057	3.5	
Dairy	Barn	-	+	-	-	9.4 (0.97)	2.7	32	
Dairy	Barn	+	-	-	-	3.2 (0.51)	1.1	9.5	
Dairy	Barn	+	-	-	-	0.9 (-0.05)	0.19	4.2	
Dairy	Barn	-	+	-	-	0.9 (-0.05)	0.19	4.2	
Dairy	Barn	+	-	-	-	0.089 (-1.05)	0.009	0.89	
Dairy	Barn	+	-	+	-	0.089 (-1.05)	0.009	0.89	
		geometric mean				3.37 (0.53)			

*MPN g-^1^ (log MPN g-^1^)

**LL-lower limit; **UL-upper limit, 95% confidence interval for MPN g-^1^ values

### Presence of *stx*, *rfbE* based on sample temperatures and depth

Pile, screened solids, and barn samples were aggregated to determine the influence of temperature on *stx* and *rfbE* presence in solid-base samples (pile, screened solids, barn). There was a significant difference in *rfbE* presence based on subsurface sample temperatures (*P* = 0.0075), with *rfbE*+ and *rfbE*- samples occurring at an average of 26.5°C and 31.1°C, respectively. There was no significant difference in *stx* presence based on subsurface sample temperatures (*P* = 0.77). There was a significant difference in *rfbE* presence based on lagoon sample temperatures (*P* = 0.047), with *rfbE*+ and *rfbE*- samples averaging 24.9°C and 25.9°C, respectively. There was no significant difference in *stx* presence based on subsurface sample temperatures (*P* = 0.91).

There was a significant difference in *stx* presence based on solid-base sample depth (*P* = 0.027), with *stx*+ and *stx*- samples occurring at an average depth of 6.5 and 17.7 cm, respectively. There was no significant difference in *rfbE* presence based on solid-base sample depth (*P* = 0.37). In lagoon samples, there was no significant difference in *stx* (*P* = 0.38), or *rfbE* (*P* = 0.65) presence based on lagoon sample depth.

### Manure storage locations and *stx* and *rfbE*+ samples

Manure storage locations were grouped into four categories–pile (n = 196), lagoon (n = 126), barn (n = 112) and screened solids (n = 84). There was a significant difference in *stx* and *rfbE* presence based on storage location. Pile samples (6%) had significantly lower *stx* presence versus lagoon (13%) and barn (11%) samples. Pile (21%) and lagoon (27%) samples were significantly higher in *rfbE* presence in comparison to barn (9%) and screened solid (8%) samples. No significant difference in *stx1* or *stx2* presence based on storage location was observed (Table 3).

**Table 3:** Percentage of *stx*, *stx1*, *stx2* and *rfbE*+ samples based on manure storage location.

Target gene	Positives by Manure Storage Location (%)			
	Pile	Lagoon	Barn	Screened Solids
	(n = 196)	(n = 126)	(n = 112)	(n = 84)
*stx*	6a*	11b	13b	8ab
*stx1*	4a	4a	5a	1a
*stx2*	4a	10a	9a	8a
*rfbE*	21a	27a	9b	8b

*Samples with the same letter are not statistically significant across rows (P > 0.05).

Screened solid (25 surface, 59 subsurface) and pile (56 surface, 140 subsurface) storage locations were used to compare 81 surface (depth = 0 cm) and 199 subsurface (depth > 0 cm) samples. Lagoon (liquid manure storage basin) and barn samples (unmoved, unstacked manure) were not classified as surface or subsurface samples and are not included in this comparison. Of the 81 surface samples, five (6%) were *stx*+ and 16 (20%) were *rfbE*+. Of the 199 subsurface samples, 13 (6.5%) were *stx*+ and 37 (19%) were *rfbE*+. No significant differences were observed between surface and subsurface samples based on *stx*, *stx1*, *stx2*, or *rfbE* presence. Of the 518 total samples analyzed, 371 had information available on storage age. Samples were divided into age categories of 0–1 (n = 170) 2–5 (n = 53), 10–20 (n = 32) and >20 days old (n = 42). The highest *stx*, *stx1* and *stx2* percentages were seen at the 10–20 day storage age. The highest percentage for *rfbE* was seen at the 6–9 day storage age. While minor variations in significance were seen in *stx* and *stx2* percentages, no significant difference were noted for *stx1* or *rfbE* percentage based on storage age (Table 4).

**Table 4:** Percentage of *stx*, *stx1*, *stx2* and *rfbE*+ samples based on storage age.

	Positives by Storage Age (Days) (%)				
Target gene	0–1 (n = 170)	2–5 (n = 53)	6–9 (n = 74)	10–20 (n = 32)	over 20 (n = 42)
*stx*	10ab*	2ab	11ab	19a	0b
*stx1*	2a	0a	7a	9a	0a
*stx2*	9ab	2bc	10ab	16a	0c
*rfbE*	15a	15a	25a	13a	5a

*Samples with the same letter are not statistically significant across rows (*P* > 0.05).

## Discussion

This study was performed to determine the prevalence and concentration of STEC in bovine manure in the Southeastern region of the US. There was at least one *stx*+ sample detected at 11 out of the 12 (92%) farms evaluated. All 12 farms were *rfbE*+ at least once during the two farm visits. Among positive farms, a prevalence rate of 9% (46/518) for *stx1/stx2* and 19% (97/518) for *E*. *coli* O157 was observed in bovine manure samples. Previous studies have observed a wide range of prevalence rates of STEC in bovine manure [29–38]. Factors such as high levels of pathogens per gram of feces, [38], persistent shedders [39], seasonality [29], and other factors may influence STEC prevalence and concentrations, although discerning the extent of these factors on an individual basis is difficult. The prevalence of *E*. *coli* O157 in feces is likely underestimated, especially if STEC concentrations are below the LOD [40] as the detection methods utilized have different levels of sensitivity and specificity [41].

Many manure surveys do not evaluate the concentration of STEC in manure samples, and studies that do quantify STEC are often limited by the low prevalence of positive samples that can be further evaluated. In this study, low MPN g^-1^ values [geometric mean of 3.37 MPN g^-1^ (0.53 log MPN g^-1^); (n = 20)] from STEC in bovine manure were observed. Omisakin et al. [40] surveyed an abattoir in the UK and found that 7.5% of cattle feces samples (n = 586) were positive for *E*. *coli* O157, and 27 of the 44 samples (61%) had an *E*. *coli* O157 concentration below the LOD (2 log CFU g^-1^), with 4 of 44 at a concentration above 4 log CFU g^-1^. Fegan et al. [42] observed geometric means of 130 MPN g^-1^ and 13 of MPN g^-1^ of *E*. *coli* O157 in 12 grain-fed and 10 pasture-fed cattle feces samples in Australian, respectively. Fegan et al. [33] compared *E*. *coli* O157 levels between lot-fed and grass-fed cattle in Australia and observed that 67% of the total fecal samples contained below 10 MPN g^-1^, with a maximum value of 1.1 x 10^5^ MPN g^-1^. Hutchison et al. [43] found a prevalence rate of 13.2 and 9.1% *E*. *coli* O157 in fresh (n = 810) and stored (n = 429) bovine manure samples in the UK, respectively, and concentrations of 2.9 x 10^6^ and 8.6 x 10^3^ CFU g^-1^ for fresh (n = 107) and stored (n = 39) samples, respectively. Brichta-Harhay et al. [31] observed *E*. *coli* O157:H7 in feedlot cattle feces at a median of 1.6 x 10^3^ CFU g^-1^. Matthews et al. [44] postulated that fewer cattle shedding high levels of STEC may be riskier than a large number of cattle shedding lower levels. As additional MPN g^-1^ data from other studies becomes available, more insights into STEC levels in bovine manure can help predict the risks of untreated BSAAO.

The two detection techniques used in this study were the mEHEC method for recovery of *stx+* samples, and the IMS-O157 method for *E*. *coli* O157 screening. Twenty-six additional samples were determined positive for *stx1* and/or *stx2* when *E*. *coli* O157+ samples were later evaluated for *stx*. The discrepancy seen between prevalence data from mEHEC and IMS-O157 methods could be due to several reasons. Immunomagnetic separation was used for the IMS-O157 method to recover cells from the non-selective TSB enrichment, which helped remove debris and other cells from the suspension prior to streaking beads for isolation, which was not implemented for the mEHEC method. For both methods, it was assumed that if STEC was present in the initial sample, it would remain viable and grow in the presence of other natural microflora. Additionally, it was assumed that only STEC grows in the mEHEC broth, though a high number of false presumptive positive isolates grew on CHROMagarSTEC throughout the present study. However, it is possible that STEC lost the lambdoid-encoded *stx* gene(s) on their chromosome during the isolation process [45, 46].

Karch et al. [45] observed the loss of *stx* during the transfer of *stx*+ colonies to both broth and agar media, regardless of the agar type used for transfer. Among the 45 isolates with Stx+ titers and confirmation of *stx+* DNA prior to subcultivation, 15 were negative for cytotoxicity and *stx* following cultivation [45]. Similarly, Joris et al. [47] evaluated 40 *stx*+ fecal samples for *stx* integrity and observed that 12 of the 40 isolates exhibited a loss of either *stx1* or *stx2* following a single subcultivation step. The loss of *stx* during the isolation process may result in lower prevalence level estimates for STEC and should be considered. Of the 97 *E*. *coli* O157+ samples in this study, 68 (70%) were negative for *stx1* and *stx2*. Wetzel and LeJeune [46] observed 17 of 81 (21%) *stx*- *E*. *coli* O157:H7 isolates from bovine manure samples. Isolates that were *rfbE*+/*stx*- could be due to *stx* loss in the environment or during the culturing process.

Samples positive for *stx2* are an important risk factor due to their association with more severe human illness versus with *stx1* [48]. Stx2 toxin has been shown to be 1,000 times more potent than Stx1 against human renal microvascular endothelial cells [49], and are more likely to result in HUS [50]. Samples negative for *stx1* or *stx2* were further evaluated for *eae* presence which revealed several *stx*-/*eae*+ samples. Not all samples had presumptive positive colonies on each agar type, therefore not all samples could be evaluated for *eae* presence. Jay-Russell et al. [51] obtained 278 isolates from dog and coyote feces collected near the Southwestern US/Mexico border as presumptive O26, O103, O145, O157, all of which were negative for *stx1* and *stx2*. Upon further analysis, 18 isolates were identified as atypical enteropathogenic *E*. *coli* (aEPEC) that were *stx1*-/stx*2*-/*eaeA*+ [51]. A high number of aEPEC strains may lead to false positives and are still a risk factor with the potential to obtain *stx* genes [52].

Researchers implement different protocols for sample collection, preparation, isolation, and detection assays to determine STEC prevalence in manure [53]. Previous research has shown that utilizing more than one media type can improve the recovery rate of STEC [31, 54]. The genetic mechanisms that lead to certain phenotypes on selective agar is not yet elucidated, and it is well recognized that the phenotype of STEC colonies varies greatly among different types of selective agar. Cooley et al. [31] found that only 9.3% (56/599) of non-O157 STEC+ samples were identified as positive on all three agar media types evaluated (Rainbow Agar O157, CHROMagar O157, and modified sheep blood agar). The thorough sampling scheme performed by Cooley et al. [31] exemplifies the need for multiple media types to more accurately isolate and identify STEC in environmental samples. In the present study, CHROMagarwas utilized for recovery of STEC following enrichment in mEHEC broth, and numerous presumptive positive colonies were not positive for *stx1* or *stx2*.

Several researchers have reported that 81.6 to 90% of the STEC isolates evaluated in their analysis could be identified on CHROMagar STEC in pure culture [55–57], which may be lower for environmental samples due to inhibition by background microbiota. For example, inoculation studies revealed the efficacy of CHROMagarSTEC for recovery ranged from 30 to 98% depending on the stool type and isolate [58]. Considering the previous evaluations of CHROMagarSTEC, modifications and/or alternatives to all media used for environmental sampling may be necessary to further optimize manure sampling protocols.

The sample temperature and depth were recorded for each sample collected in this study. There was a significant difference in *rfbE* presence based on solid-base sample temperatures as well as lagoon sample temperatures, but no significant difference in *stx* presence based on subsurface or lagoon sample temperatures. It should be noted that although significant, a minor difference in temperature in lagoon samples based on *rfbE* presence. There was a significance in *stx* presence based on sample depth for solid-base samples. Additional data is needed to determine how storage methods can influence STEC survival. Significant differences in both *stx* and *rfbE* presence were observed based on manure storage method. Farm practices can vary widely among collection sites, which makes it even more difficult to generalize and determine a baseline prevalence in bovine manure due to differences in manure storage, treatment, and time of manure sitting undisturbed. Statistical analysis was not performed on equipment cleaning practices, as only four of 12 farmers provided information on the frequency of cleaning manure-handling equipment. Of these four farms, two cleaned equipment daily, one cleaned equipment weekly, and one cleaned equipment bi-monthly. None of the farms visited had a means of manure pile protection from birds, rodents or other pests. Equipment cleaning and segregation is an important step to limit cross contamination on the farm between the manure storage/treatment areas and the rest of the farm [59], especially if grown in close proximity to produce farms or if equipment is used near the time of harvest.

Foodborne outbreaks continue to be problematic for growers, processors, and consumers in the produce industry [2]. This research provides data on STEC prevalence and concentration in bovine manure collected from farms in Florida. Conclusions based on prevalence of *stx* and *rfbE* in bovine manure samples should also consider the available selective media that was used in an attempt to recover STEC from these environmental samples as well as the potential for *stx* loss during the isolation process. Additional data from different geographical regions will help determine STEC levels in bovine manure across the US. Ultimately, this data will be used to determine the risk associated with the application of untreated BSAAO in the PSR. In addition to following future regulations on harvest intervals, growers should be aware of the handling practices and manure management strategies and adjust harvest intervals as necessary to limit contamination of produce from untreated BSAAO.

## Supporting information

S1 File S1_Bovine manure data stx_rfbE. (XLSX)
